# Pre-operative Cerebral Small Vessel Disease on MR Imaging Is Associated With Cerebral Hyperperfusion After Carotid Endarterectomy

**DOI:** 10.3389/fcvm.2021.734392

**Published:** 2021-11-18

**Authors:** Xiaoyuan Fan, Zhichao Lai, Tianye Lin, Hui You, Juan Wei, Mingli Li, Changwei Liu, Feng Feng

**Affiliations:** ^1^Department of Radiology, Peking Union Medical College Hospital, Chinese Academy of Medical Sciences and Peking Union Medical College, Beijing, China; ^2^Department of Vascular Surgery, Peking Union Medical College Hospital, Chinese Academy of Medical Sciences and Peking Union Medical College, Beijing, China; ^3^General Electric Healthcare, MR Research China, Beijing, China; ^4^State Key Laboratory of Difficult, Severe and Rare Diseases, Peking Union Medical College Hospital, Chinese Academy of Medical Sciences and Peking Union Medical College, Beijing, China

**Keywords:** cerebral small vessel disease, carotid stenosis, carotid endarterectomy, hyperperfusion syndrome, magnetic resonance imaging

## Abstract

**Objectives:** To determine whether pre-operative cerebral small vessel disease is associated with cerebral hyperperfusion (CH) after carotid endarterectomy (CEA).

**Methods:** Seventy-seven patients (mean age of 66 years and 58% male) undergoing CEA for carotid stenosis were investigated using brain MRI before and after surgery. CH was defined as an increase in cerebral blood flow > 100% compared with pre-operative values on arterial spin labeling MR images. The grade or the number of four cerebral small vessel disease markers (white matter hyperintensities, lacunes, perivascular spaces, and cerebral microbleeds) were evaluated based on pre-operative MRI. Cerebral small vessel disease markers were correlated with CH by using multivariate logistic regression analysis. The cutoff values of cerebral small vessel disease markers for predicting CH were assessed by receiver-operating characteristic curve analysis.

**Results:** CH after CEA was observed in 16 patients (20.78%). Logistic regression analysis revealed that white matter hyperintensities (OR 3.09, 95% CI 1.72–5.54; *p* < 0.001) and lacunes (OR 1.37, 95% CI 1.06–1.76; *p* = 0.014) were independently associated with post-operative CH. Receiver-operating characteristic curve analysis showed that Fazekas score of white matter hyperintensities ≥3 points [area under the curve (AUC) = 0.84, sensitivity = 81.3%, specificity = 73.8%, positive predictive value (PPV) = 44.8% and negative predictive value (NPV) = 93.8%] and number of lacunes ≥ 2 (AUC = 0.73, sensitivity = 68.8%, specificity = 78.7%, PPV = 45.8% and NPV = 90.6%) were the optimal cutoff values for predicting CH.

**Conclusion:** In patients with carotid stenosis, white matter hyperintensities and lacunes adversely affect CH after CEA. Based on the NPVs, pre-operative MR imaging can help identify patients who are not at risk of CH.

## Introduction

Carotid endarterectomy (CEA) is an established procedure for the prevention of further ischemic events caused by carotid stenosis. However, post-operative complications may reduce the benefits of surgery. Cerebral hyperperfusion syndrome (CHS), characterized by severe throbbing headache, confusion, seizures, focal neurological deficits and occasional intracranial hemorrhage, is associated with a mortality rate of 38.2% and permanent disability of 28% (1). CHS often occurs in patients with cerebral hyperperfusion (CH), which is defined as an increase in perfusion by >100% after surgery compared with baseline (2). If not recognized and treated early, a subset of CH-patients may further develop CHS. Impaired cerebral autoregulation is the most accepted mechanism for CH (3). After carotid revascularization, impaired cerebral autoregulation cannot maintain a stable cerebral blood flow (CBF) *via* constriction of cerebral arterioles and capillaries in response to a sudden increase in cerebral perfusion pressure, which leads to CH.

From the perspective of the pathogenesis of CH, pre-operative cerebral small vessel disease (SVD) may adversely affect CH after CEA (2). Cerebral SVD is a disorder of the cerebral arterioles and capillaries with cerebral small vessel endothelial dysfunction being the major pathological mechanism. Endothelial damage leads to the limitation of vasomotor function of cerebral small vessels, and then impaired cerebrovascular autoregulation ability (4). Common cerebral SVD lesions on magnetic resonance imaging (MRI) include white matter hyperintensity (WMHs), lacunes, perivascular spaces (PVSs), and cerebral microbleeds (CMBs) (5). A previous study (6) found in patients with carotid occlusion, ipsilateral WMHs were specific and sensitive for the presence and severity of decreased cerebrovascular reserve, which is an important manifestation of cerebral autoregulation (7) and recommended as the gold standard for predicting CH (8). This study also gave a hint that pre-operative cerebral SVD may be associated with CH after CEA. However, the relationship between cerebral SVD and CH has not been confirmed until now.

The common cerebral SVD lesions on MRI are reliable and easy to collect as long as the standardized definitions are adopted (5); thus, pre-operative cerebral SVD may be useful imaging markers for the prediction of CH that can be applied in clinical practice. This study aimed to investigate whether pre-operative cerebral SVD was associated with CH after CEA, and to exhibit practical cutoff values of cerebral SVD markers for predicting CH. In a group of patients undergoing CEA for carotid stenosis, we evaluated the relationship between cerebral SVD and CH using a combination of MRI methods including conventional structural imaging and perfusion weighted imaging.

## Materials and Methods

### Study Design and Patients

This prospective, single-center observational study was approved by the Medical Ethics Committee of the Peking Union Medical College Hospital, in line with the Declaration of Helsinki. All participants provided written informed consent for this study.

**Figure 1:**
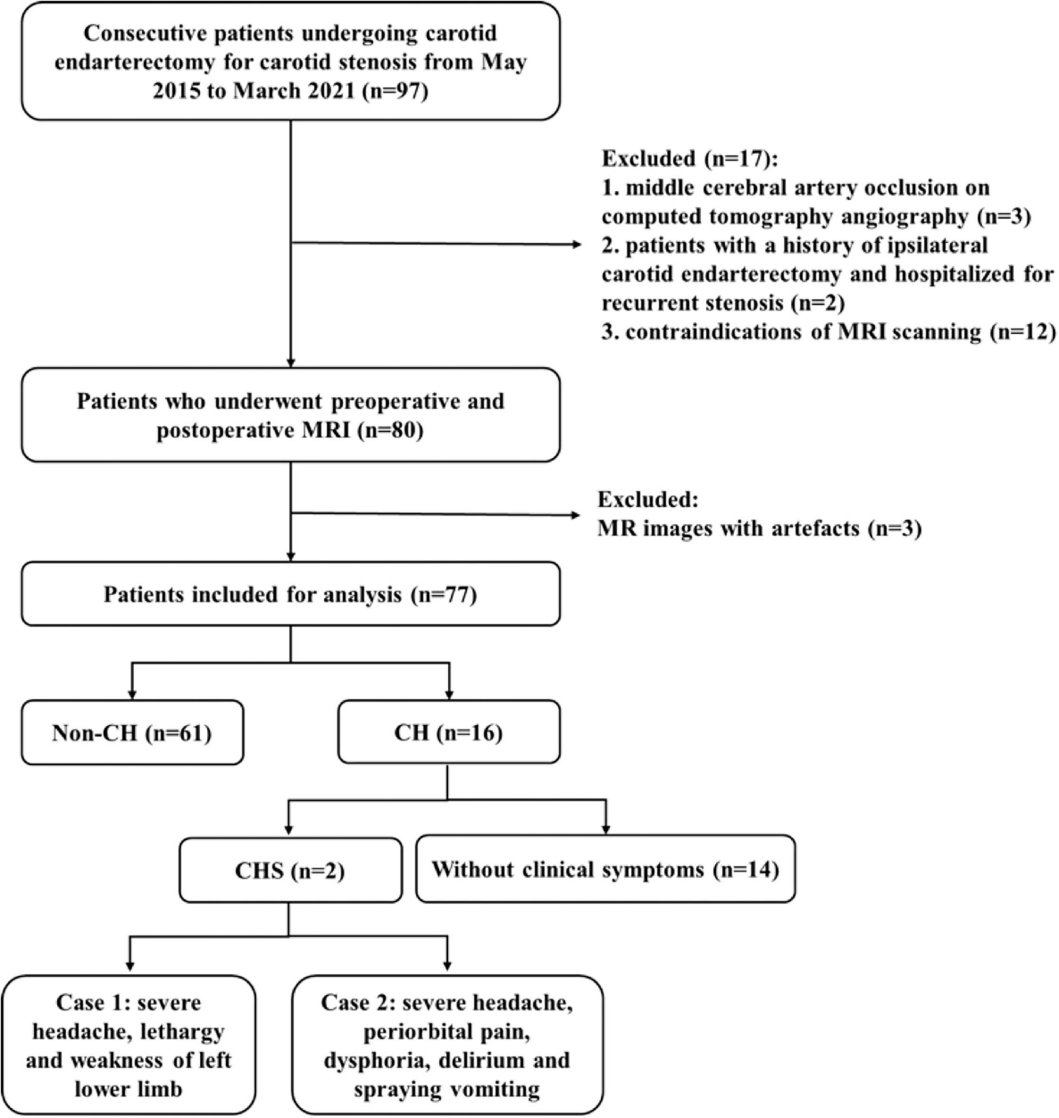
Flowchart of patient enrollment.

We consecutively enrolled patients who underwent CEA for unilateral or bilateral carotid stenosis [≥50% for symptomatic stenosis or ≥70% for asymptomatic stenosis, according to the North American Symptomatic Carotid Endarterectomy Trial (NASCET) grading (9)] diagnosed with computed tomography angiography. The exclusion criteria included: (1) patients with intracranial artery stenosis ≥50% or occlusion shown by pre-operative computed tomography angiography, (2) history of ipsilateral CEA and re-admission due to carotid re-stenosis, (3) contraindications of MRI scanning or refuse MRI scanning, or (4) artifacts on MR images that interfere with evaluation. Pre-operative MRI was obtained within 2 weeks before CEA and post-operative MRI was obtained within 7 days after CEA. From May 2015 to March 2021, 97 patients were initially included in this study. Three patients had ipsilateral middle cerebral artery occlusion, 2 patients had a history of ipsilateral CEA, 12 patients had contraindications of MRI scanning and 3 patients had artifacts on MRI. A final 77 patients were enrolled in our study. A flow chart of the patient enrollment is shown in Figure 1.

### MRI

All MRI examinations were performed on a 3.0 T scanner (Discovery 750, GE Healthcare) with an eight-channel phased-array head coil. Standard pseudo-continuous arterial spin labeling (ASL) was performed with a 3D stack-of-spirals fast-spin-echo readout: labeling duration/post labeling delay = 1,450/2,025 ms, TR/TE = 4,886/10.5 ms, in-plane spiral number 8, points per spiral 512, field of view (FOV) = 240 mm × 240 mm, in-plane resolution 3.75 mm × 3.75 mm, 40 slices and slice thickness = 4 mm. CBF maps of standard ASL were generated on GE AW 4.5 workstation by a commercial software 3D ASL Functool kit. Conventional MRI sequences including diffusion-weighted imaging, T1-weighted, T2-weighted, fluid-attenuated inversion recovery, and T2^*^-weighted gradient-recalled echo imaging were also performed (Supplementary Table 1).

### Evaluation of Cerebral SVD

Four imaging markers of cerebral SVD on MRI including WMHs, lacunes, PVSs, and CMBs were recorded according to the previously reported neuroimaging standards (5). Table 1 shows the detailed evaluation criteria of four MRI markers and the total cerebral SVD burden score. Briefly, WMHs were graded using the Fazekas score ranged from 0 to 6 by summing the deep and periventricular WMH scores (10). The number of lacunes located in the territory of a perforating arteriole was conservatively counted. The number of CMBs not strictly located in lobes was also recorded. We graded the number of PVSs in the basal ganglia with a three-category ordinal scale as follows: 0–10 (category 1), 11–25 (category 2), and >25 (category 3) (10). For each patient, an overall cerebral SVD burden score was calculated according to the presence of each cerebral SVD marker (11). The overall cerebral SVD score ranged from 0 to 4.

**Table 1:** Evaluation criteria of the four cerebral SVD markers and the total cerebral SVD burden score.

**MRI markers**	**Definition and grades**	**Number or degree**	**Score**
WMHs	WMHs were defined as hyperintensity on T2-weighted images and fluid-attenuated inversion recovery without cavitation. Periventricular WMHs were graded as: 0 = absent, 1 = caps or pencil-thin lining, 2 = smooth halo, or 3 = irregular periventricular WMHs extending into the deep white matter. Deep WMHs were graded as: 0 = absent, 1 = punctate foci, 2 = beginning confluent foci, or 3 = large confluent areas.	Fazekas score <2 in the deep white matter and <3 in the periventricular white matter Fazekas score ≥2 in the deep white matter or ≥3 in the periventricular white matter	0 1
Lacune	Lacune was defined as a round or ovoid, subcortical, fluid-filled cavity of between 3 mm and about 15 mm in diameter located in the territory of a perforating arteriole. Number of lacunes was conservatively counted.	0 ≥1 lesion	0 1
CMBs	CMBs were defined as a small (generally 2–5 mm in diameter) area of signal void with associated blooming seen on T2*-weighted MRI. Number of CMBs were counted. CMBs strictly located in lobes were not recorded.	0 ≥1 lesion	0 1
PVSs	PVSs were round or ovoid, with a diameter generally smaller than 3 mm and had signal intensity similar to cerebrospinal fluid on all sequences. The number of PVSs in the basal ganglia was graded with a three-category ordinal scale as follows: 0–10 (category 1), 11–25 (category 2), and >25 (category 3).	Category-1 PVSs Category-2 or−3 PVSs	0 1

*SVD, small vessel disease; MRI, magnetic resonance imaging; WMHs, white matter hyperintensity; CMBs cerebral microbleeds; PVSs, perivascular spaces*.

Two neuroradiologists (H. You and T. Lin, with 19 and 7 years of neuroradiology experience, respectively) were trained before evaluation and assessed the MRI data blindly for clinical information. Disagreements were resolved by consensus.

### Diagnosis of CH and CHS

Regions of interest were set at the cerebral cortex perfused by ipsilateral carotid artery in watershed areas and 8 perfusion territories (2 anterior and 6 middle cerebral artery territories), corresponding with the Alberta Stroke Programme Early Computed Tomography Score locations (12). The region of interest placement was consistent between pre-operative and post-operative CBF images. CH was defined as an increase in CBF >100% compared with pre-operative values in ≥1 regions of interest with or without clinical symptoms or signs (2).

CHS was defined as: (1) existence of CH, (2) occurrence of a throbbing frontotemporal or periorbital headache on the ipsilateral side of the CEA, seizure, confusion, deterioration of consciousness level, development of focal neurological signs, or intracranial hemorrhage, and (3) absence of new ischemic lesions on post-operative MRI (2).

### Other Clinical and Imaging Characteristics

The presence of symptoms and time elapsed since the last cerebrovascular event were recorded by an experienced vascular surgeon. Age, sex, and vascular risk factors including hypertension, diabetes mellitus, dyslipidemia, coronary heart disease, history of smoking and alcohol were collected, according to self-reported history, medication history or referring to the results of laboratory examination. The use of intraluminal shunt during surgery, baseline systolic blood pressure (BP) on admission, the highest systolic BP within 24 h after CEA and the highest systolic BP from the second day after CEA to discharge were also recorded. The degree of stenosis was determined according to the NASCET criteria (9) by computed tomography angiography. We carefully performed the diagnosis of carotid near-occlusion by using an interpretive approach based on previous studies (13, 14).

### Perioperative and Post-operative Management

CEA was performed under general anesthesia. A bolus of heparin (100 U/kg) was administered intravenously for systemic anticoagulation. All CEAs were carefully performed by vascular surgeons with more than 10 years of experience. Carotid atherosclerotic plaques were removed by classic longitudinal arteriotomy with patching or eversion surgery. If patients were observed with poor collateral circulation before surgery (15) or with asymmetry or diffuse slowing of the electroencephalogram during clamping (16), an intraluminal shunt was used. Following surgery, patients were transferred to the post-anesthesia care unit for about 1 h until the blood pressure (BP) was stable, before transfer back to the vascular ward.

Patients received electrocardiogram monitoring for 1–2 days after surgery. BP was closely monitored to control systolic BP between 120 and 140 mmHg. BP was measured three times daily using a sphygmomanometer after removal of electrocardiogram monitoring. Patients with a systolic BP > 140 mmHg received oral antihypertensive drugs as the first-line treatment. If systolic BP remains elevated, intravenous antihypertensive drugs were given. For patients who complained of CHS symptoms, intravenous mannitol or glycerol fructose was added to lower intracranial pressure.

### Statistical Analysis

All data was analyzed using statistical software (IBM SPSS v25.0). The κ value was calculated for the inter-observer agreement of each cerebral SVD marker. The κ values ≤ 0.40 represented poor agreement, values > 0.40 and ≤ 0.65 represented general agreement, values > 0.65 and ≤ 0.75 represented good agreement, and values > 0.75 represented excellent agreement. The relationship between each variable and CH was analyzed by univariate analysis. To determine the association of cerebral SVD with CH adjusted for other risk factors, we performed multivariate logistic regression analysis by using a forward stepwise method. Age, sex and variables with *p* < 0.2 in univariate analysis were entered into the logistic regression models as covariables. Since SVD markers share the common pathogenesis and often coexist, to avoid the risk of multicollinearity, each cerebral SVD marker and the total cerebral SVD score entered different regression models, respectively, rather than including them in the one model. The remaining covariates entered all models consistently. The optimal cutoff values of cerebral SVD markers for predicting CH were assessed using receiver-operating characteristic (ROC) curve analysis. The sensitivity, specificity, and negative and positive predictive values of cerebral SVD markers for differentiating patients with and without CH were calculated. All *p*-values were calculated using two-tailed tests. A value of *p* < 0.05 was considered statistically significant.

## Results

### Characteristics of the Study Population

CEA was successfully performed in all patients. Of the 77 patients, 16 (20.78%) met CBF criteria for CH. The clinical and radiological characteristics of the study population are shown in Table 2. The mean age was 66 ± 7 years and 75.3% were males. Of the 77 patients, 14 (18.2%) patients had transient ischemic attack and 16 (20.8%) patients had ischemic stroke. No emergency surgery was performed. The median Fazekas score was 2 points and median PVSs category was 1 grade. Thirty-eight (49.4%) patients had lacunes and 20 (25.3%) patients had CMBs.

**Table 2:** Clinical characteristics and cerebral small vessel disease markers of patients.

	**All (*n* = 77)**	**CH (*n* = 16)**	**Non-CH (*n* = 61)**	***p***
Age, years	66.0 ± 7.3	66.4 ± 8.5	65.8 ± 7.0	0.765
Male	58 (75.3)	15 (93.8)	43 (70.5)	0.099
Presence of symptoms				0.324
Asymptomatic stenosis	47 (61)	7 (43.8)	40 (65.6)	
TIA	14 (18.2)	4 (25)	10 (16.4)	
Stroke	16 (20.8)	5 (31.3)	11 (18)	
Days since the last cerebrovascular event	42.5 [27, 75]	45 [20, 120]	40 [33, 60]	0.304
Large infarcts on MRI^a^	15 (19.5)	6 (37.5)	9 (14.8)	0.071
Hypertension	53 (68.8)	13 (81.3)	40 (65.6)	0.364
Diabetes	28 (36.4)	7 (43.8)	21 (34.4)	0.490
Dyslipidemia	37 (48.1)	7 (43.8)	30 (49.2)	0.783
Coronary artery disease	17 (22.1)	6 (37.5)	11 (18.0)	0.172
Smoking	41 (53.2)	10 (62.5)	31 (50.8)	0.405
alcohol	22 (28.6)	3 (18.8)	19 (31.1)	0.375
Ipsilateral stenosis				*0.002* ^*^
50–70%	10 (13)	0 (0)	10 (16.4)	
70–99%	52 (67.5)	8 (50)	44 (72.1)	
Near-occlusion	15 (19.5)	8 (50)	7 (11.5)	
Contralateral stenosis				0.608
0	26 (33.8)	5 (31.3)	21 (34.4)	
<50%	34 (44.2)	7 (43.8)	27 (44.3)	
50–70%	8 (10.4)	1 (6.3)	7 (11.5)	
70–99%	7 (9.1)	3 (18.8)	4 (6.6)	
Occlusion	2 (2.6)	0 (0)	2 (3.3)	
Shunt use	49 (63.6)	11 (68.8)	38 (62.3)	0.633
BP_baseline, mmHg	136.7 ± 15.7	140.7 ± 17.9	135.7 ± 15	0.258
BP_post_1st day, mmHg	126.0 ± 14.9	130.5 ± 20.7	124.8 ± 12.9	0.308
BP_before discharge, mmHg	139.4 ± 14.2	139.9 ± 21.1	139.3 ± 12.0	0.933
Cerebral SVD markers				
Total SVD score	1 [0–2]	2 [1.25–3]	1 [0–2]	*0.001* ^†^
0	23 (29.9)	1 (6.3)	22 (36.1)	
1	25 (32.5)	3 (18.8)	22 (36.1)	
2	15 (19.5)	7 (43.8)	8 (13.1)	
3	10 (13)	3 (18.8)	7 (11.5)	
4	4 (5.2)	2 (12.5)	2 (3.3)	
Fazekas score of WMHs	2 [1–3]	4 [3–5]	1 [1–3]	*<0.001* ^†^
Any lacunes	38 (49.4)	12 (75)	26 (42.6)	*0.021* ^*^
Number of lacunes	0 [0–2]	2.5 [0.25–4.75]	0 [0–1]	*0.003* ^†^
Category of PVSs	1 [1–2]	1 [1–2]	1 [1–1.5]	0.198
Any CMBs	20 (25.3)	5 (31.3)	15 (24.6)	0.749
Number of CMBs	0 [0–1]	0 [0–1]	0 [0–0.5]	0.492

*CH, cerebral hyperperfusion; TIA, transient ischemic attack; BP_baseline, baseline systolic blood pressure on admission; BP_ post_1st day, the highest systolic blood pressure within 24 h after surgery; BP_before discharge, the highest systolic blood pressure from the second day after surgery to discharge; SVD, small vessel disease; WMHs, white matter hyperintensities; PVSs, perivascular spaces; CMBs, cerebral microbleeds*.

*Normally distributed continuous variables were expressed as means ± standard deviation. Categorical variables were expressed as percentages of patients who satisfied the criteria. Discrete variables were expressed as median [interquartile range]*.

a *Cortical infarcts or subcortical hemispheric infarcts >1.5 cm in diameter within the territory of ipsilateral carotid artery on MRI*.

* *Chi-square test or Fisher's exact test*.

† *Mann-Whitney U test*.

*Italic values mean p < 0.05 between CH and non-CH groups*.

### Inter-observer Agreement

Inter-observer agreement for each cerebral SVD marker was good or excellent (Fazekas score, κ = 0.78; number of lacunes, κ = 0.72; PVSs category: κ = 0.77; number of CMBs, κ = 0.86).

### Relationship Between Cerebral SVD and CH

The results of univariate analysis between CH and non-CH group are shown in Table 1. The total cerebral SVD score was significantly higher in patients with CH compared with patients without CH (*p* = 0.001). As for the four cerebral SVD markers, the Fazekas score (*p* < 0.001), presence of lacunes (*p* = 0.021) and number of lacunes (*p* = 0.003) were associated with CH after CEA. There were no significant differences in PVSs or CMBs between the two groups (*p* = 0.198 and *p* = 0.749, respectively). In terms of clinical characteristics, patients with CH had a significantly higher degree of ipsilateral carotid stenosis than those without CH (*p* = 0.002). Patients with carotid near-occlusion had the highest risk of CH after CEA (Supplementary Figure 1). No statistical difference was found in other clinical variables.

Age, gender, large infarcts on MRI, coronary artery disease and degree of ipsilateral carotid stenosis were included as covariates according to the result of univariate analysis. In the logistic regression models, Fazekas score of WMHs [OR 3.09, 95% CI (1.72–5.54); *p* < 0.001], number of lacunes [OR 1.37, 95% CI (1.06–1.76); *p* = 0.014] and total cerebral SVD score [OR 2.68, 95% CI (1.42–5.07); *p* = 0.002] were still significantly associated with post-operative CH (Table 3). No association was found between PVSs and CH (*p* = 0.184). Ipsilateral degree of stenosis was associated with CH in all regression models (*p* < 0.05). No statistical difference was observed between CH and other variables.

**Table 3:** Logistic regression analysis of relationships between cerebral SVD markers and post-operative cerebral hyperperfusion.

**Cerebral SVD markers**	***p***	**OR**	**95% CI**
WMHs	<0.001	3.09	1.72–5.54
Number of lacunes	0.014	1.37	1.06–1.76
PVSs	0.184	NA	NA
Total cerebral SVD score	0.002	2.68	1.42–5.07

*SVD, small vessel disease; WMHs, the Fazekas score of white matter hyperintensities; PVSs, category of perivascular spaces*.

### ROC Curves Analysis

**Figure 2:**
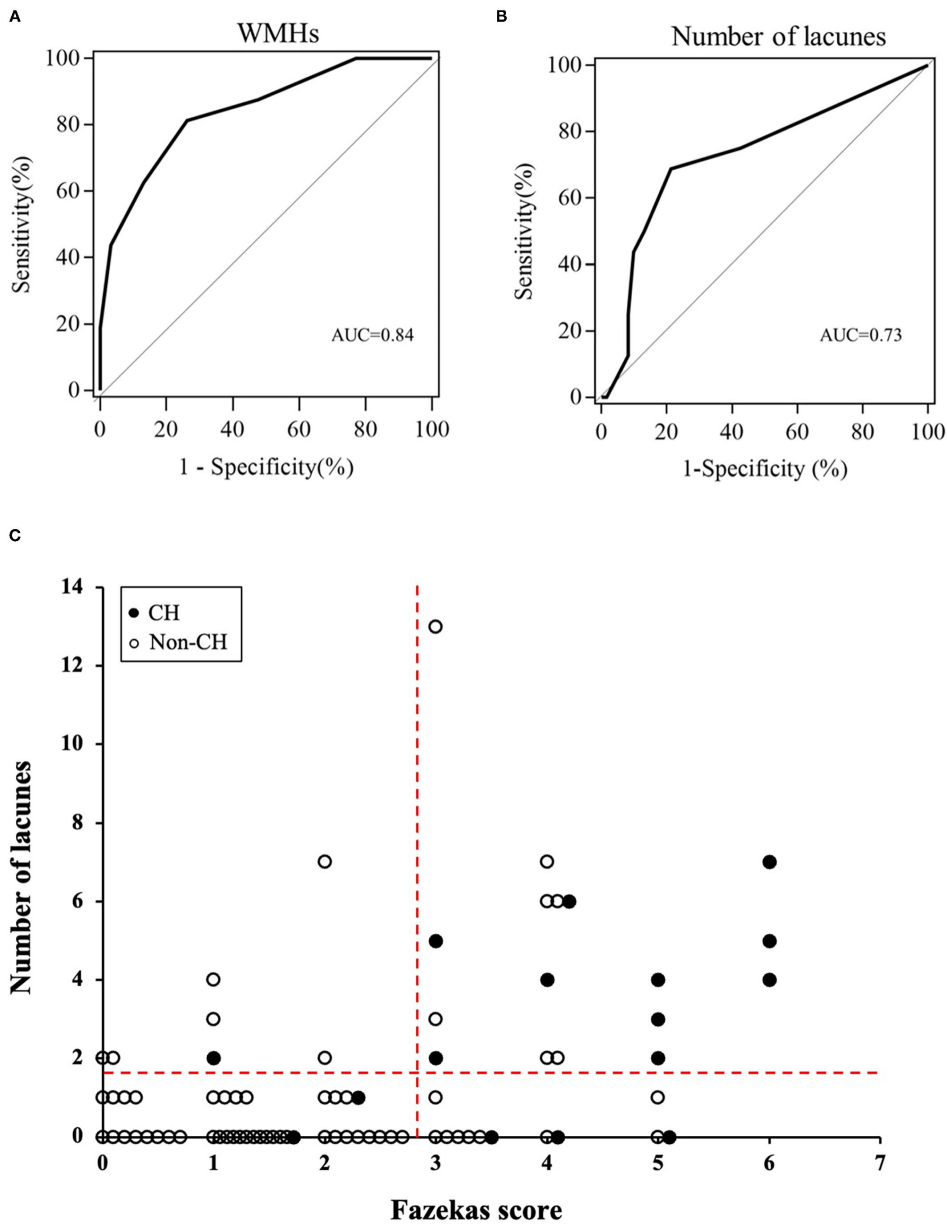
The receiver operating characteristic (ROC) curves of pre-operative Fazekas score **(A)** and number of lacunes **(B)** for the prediction of cerebral hyperperfusion (CH) after surgery. **(C)** Relationships between the Fazekas score, number of lacunes, and post-operative CH. The dotted horizontal line denotes the cutoff value of lacunes (≥2) obtained from the ROC curve for prediction of CH. The dotted vertical line denotes the cutoff value of Fazekas score (≥3) obtained from the ROC curve for prediction of CH.

**Figure 3:**
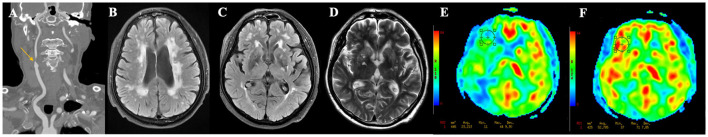
A representative case of cerebral hyperperfusion. **(A)** Pre-operative computed tomography angiography shows severe stenosis of the right carotid artery. **(B–D)** Pre-operative T2WI and FLAIR show severe white matter hyperintensities (Fazekas score = 6), multiple lacunes in bilateral basal ganglia, and category-3 perivascular spaces. **(E)** Pre-operative arterial spin labeling image shows a significantly decreased cerebral blood flow (23.21 ml/100 g/min) in the watershed area. **(F)** Post-operative arterial spin labeling image shows the cerebral blood flow (52.79 ml/100 g/min) increase >100% compared with pre-operative values.

Fazekas score ≥ 3 points [area under the curve (AUC) = 0.84, sensitivity = 81.3%, specificity = 73.8%, positive predictive value (PPV) = 44.8% and negative predictive value (NPV) = 93.8%] and number of lacunes ≥ 2 (AUC = 0.73, sensitivity = 68.8%, specificity = 78.7%, PPV = 45.8% and NPV = 90.6%) were the optimal cutoff values for the prediction of post-operative CH (Figures 2A,B). The relationships between the Fazekas score, the number of lacunes and CH are shown in Figure 2C. Of the 41 patients with a Fazekas score <3 and the number of lacunes <2, only 2 (4.88%) patients had CH after CEA. And importantly, both the 2 patients had ipsilateral carotid near-occlusion. A representative case of CH is shown in Figure 3.

### Patients Diagnosed With CHS

Of the 16 patients with post-CEA CH, 2 developed CHS. One patient diagnosed with CHS complained of an ipsilateral severe throbbing headache, lethargy, and weakness of the left lower limb. Another CHS patient had an ipsilateral severe throbbing headache, periorbital pain, dysphoria, delirium and spraying vomiting. After strict control of BP and reduction of intracranial pressure, further intracranial hemorrhage did not occur.

**Figure 4:**
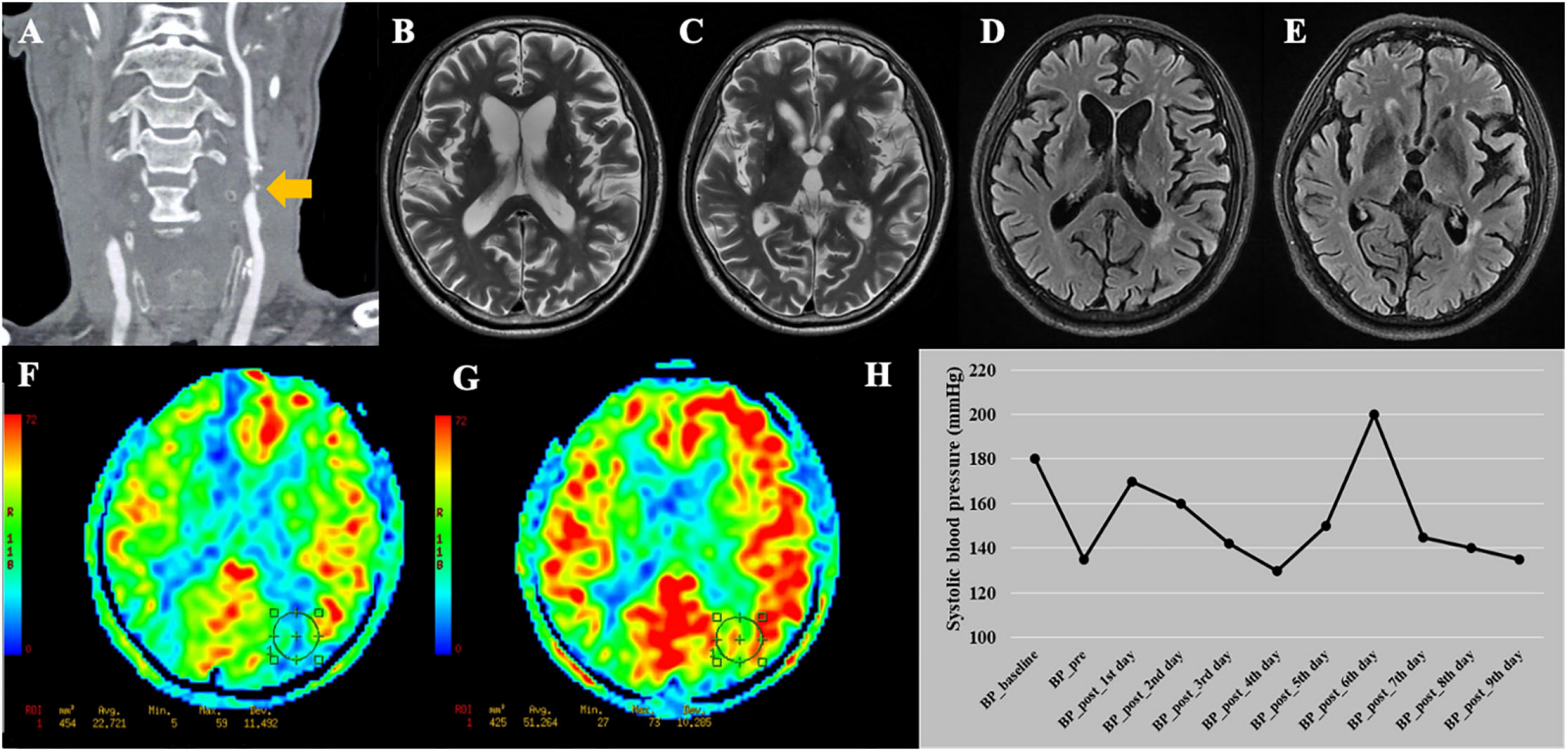
A representative case of cerebral hyperperfusion syndrome. **(A)** This patient was diagnosed with bilateral severe carotid stenosis. During this hospitalization, this patient received carotid endarterectomy (CEA) on the left side. **(B–E)** Pre-operative T2WI and FLAIR show Fazekas score of 4 points and multiple lacunes in bilateral basal ganglia and left thalamus. **(F)** Pre-operative arterial spin labeling image shows a significantly decreased cerebral blood flow (22.72 ml/100 g/min) in the watershed area. **(G)** Post-operative arterial spin labeling image shows the cerebral blood flow (51.26 ml/100 g/min) increase >100% compared with pre-operative values. **(H)** This patient developed cerebral hyperperfusion syndrome during the first day after CEA with systolic blood pressure of 170 mmHg. The patient experienced dysphoria, delirium under fluctuating blood pressure. After adjusting the medication, the patient discharged with stable blood pressure. BP_baseline, baseline systolic blood pressure on admission; BP_pre, systolic blood pressure measured the day before surgery; BP_post, systolic blood pressure measured everyday after CEA before discharge.

The basic data of the 16 patients are listed in Supplementary Table 2. Both of the 2 CHS patients were male, had coronary artery disease, and showed clinical symptoms before surgery. Moreover, the two CHS patients showed a trend toward a higher maximum systolic BP within 24 h after surgery (160 and 170 mmHg) compared with the other 14 patients (mean value = 126 mmHg). A representative case of CHS is shown in Figure 4.

## Discussion

As a large artery atherosclerotic disease, the role of cerebral SVD was less mentioned in patients with carotid stenosis. Actually, carotid stenosis often coexists with cerebral SVD because they share common systematic vascular risk factors such as aging and hypertension (17). In this preliminary study, we explored the relationship between cerebral SVD MRI markers and CH after CEA in patients with carotid stenosis and we found that pre-operative WMHs and lacunes adversely affected CH after CEA. This is, to the best of our knowledge, the first study to examine the association between cerebral SVD and CH after CEA in patients with carotid stenosis.

CHS is a serious perioperative complication after CEA and a major cause of intracerebral hemorrhage during hospitalization (18). The North American Symptomatic Endarterectomy Trial (19) demonstrated that WMHs were associated with a higher risk of any stroke (non-fatal or fatal ischemic stroke and hemorrhagic stroke confirmed by brain imaging) within 30 days after CEA. However, that study did not report the rate of ischemic and hemorrhagic stroke separately or the presence of CH or CHS. Our findings showed that pre-operative WMHs and lacunes were independently associated with post-operative CH. A possible mechanism for this association involves impaired cerebrovascular autoregulation, which is known as the main mechanism of CH (2, 3). WMHs and lacunes are common MRI markers for cerebral SVD caused by cerebral microvascular endothelial dysfunction (4). Patankar et al. reported that even in the presence of severe stenotic/occlusive large vessel disease, microvascular abnormalities are the predominant pathogenetic factor in WMHs (20). With increasing WMH burden and lacunes, permeability of the small vessel wall increases, followed by inflammatory reaction, thickening and stiffness of the vessel wall, and impaired cerebral autoregulation ability (15). Another possible mechanism may be the free oxygen radicals. Patients with cerebral SVD would suffer from cerebrovascular oxidative stress and extensive reactive oxygen species production (21), which can cause vasodilation and increased permeability of cerebral vessels during ischemia reperfusion (2).

The NPVs of Fazekas score ≥ 3 points and number of lacunes ≥ 2 were >90%. In our study, none of patients with both Fazekas score < 3 points and number of lacunes < 2 (*n* = 41) experience CH, except for 2 patients with carotid near-occlusion. These findings indicate that combining the information of WMHs, lacunes (representing the influence of cerebral small vessels) and degree of stenosis (representing the influence of extracranial large arteries) may identify patients who would not develop CH after CEA. This can obviate unnecessary invasive intravenous antihypertensive therapy on many post-CEA patients, different from the one-size-fits-all strategy that treating all patients with systolic BP > 140 or 160 mmHg (3, 22). Moreover, it will help clinicians decide who can safely return to ward or home instead of staying in intensive care unit; thus, this will lead to a reduction in hospital and patient costs.

Furthermore, all 6 patients with both a Fazekas score of 5 or 6 and number of lacunes ≥2 (2 of them with carotid near-occlusion, Supplementary Table 2) developed CH after CEA. Thus, our results gave a hint that clinicians may need to pay particular attention for those patients with both a higher Fazekas score (5 or 6 points) and more lacunes (≥2) during the surgical and perioperative periods, such as use of shunt or shortening of the cross-clamping time, more careful use of anticoagulants and antiplatelet therapy, and strict control of BP to minimize the occurrence of CH/CHS (2). Given the relatively small number of patients with severe WMHs (Fazekas score of 5 or 6, 9 of 77), this finding needs to be verified by future studies with a larger sample size.

In the present study, the incidence of CHS after CEA was 2.6%, and 12.5% of patients with CH developed CHS, which was consistent with previous reports (23, 24). Of the 16 CH patients, the 2 patients who further developed CHS had a higher systolic BP within 24 h after CEA than the remaining 14 patients. Post-operative hypertension has been demonstrated as an important risk factor for CHS by previous studies, both in CEA and CAS (1–3, 25, 26). The current study emphasized the effect of early post-operative BP during the progression from CH to CHS. Moulakakis et al. (27), Newman et al. (28) reported that post-endarterectomy hypertension was associated with higher post-operative pain scores, which may partially explain our findings. However, because of the small number of CHS patients, the mechanism of BP in CHS requires further exploration. Anyway, strict control of BP in the perioperative phase is essential in the prevention and management of CHS (2).

The study patients received CEA under general anesthesia. CEA is the standard treatment for patients with symptomatic carotid stenosis, and general anesthesia is the most preferable technique for CEA practiced by clinicians (29–31). Our findings may be applicable to most clinical situations. Theoretically, impaired cerebral autoregulation is a shared mechanism of CH for both CEA and CAS; thus, our finding of the association between cerebral SVD and CH may also apply to CAS. Some of general anesthetics may influence cerebral hemodynamics by vasodilating effects or disturbing blood flow-activity coupling (2, 32), while anesthesia protocols that may disturb CBF and autoregulation were not performed in the current study. Performing CEA under regional anesthetics does not impair cerebral hemodynamic, which may be a better choice for CH/CHS studies.

There are several limitations to our study. First, the sample size was relatively small for the low incidence of CHS. Since only 2 patients presented with CHS, statistical analysis was not performed for CHS patients. Our preliminary findings of the cutoff values of cerebral SVD for the prediction of CH need to be validated by further studies with larger sample sizes. Second, cerebrovascular reserve was not performed in our study. The pathogenesis underlying the relationship between cerebral SVD and CH cannot be directly verified in our study. Actually, cerebrovascular reserve studies are in progress in our unit. Third, our conclusions are applicable to patients undergoing CEA under general anesthesia, but cannot be directly extrapolated to patients undergoing carotid artery stenting or to those under local anesthesia. Whether our findings are applicable for CAS or CEA under regional anesthesia needs to be verified by future studies.

## Conclusion

In conclusion, pre-operative WMHs and lacunes adversely affect CH after CEA. In patients with carotid stenosis, cerebral SVD may be an important risk factor for CH after CEA and can serve as a useful imaging marker for CH, especially for predicting patients who will not develop CH. When considering the risk of CH in patients undergoing CEA for carotid stenosis, pre-operative brain MRI could help clinicians make treatment decisions.

## Data Availability Statement

The raw data supporting the conclusions of this article will be made available by the authors, without undue reservation.

## Ethics Statement

The studies involving human participants were reviewed and approved by the Medical Ethics Committee of the Peking Union Medical College Hospital. The patients/participants provided their written informed consent to participate in this study. Written informed consent was obtained from the individual(s) for the publication of any potentially identifiable images or data included in this article.

## Author Contributions

XF and TL: conception, design, and statistical analysis. XF, ZL, ML, and FF: analysis, interpretation, and final approval of the article. XF, ZL, TL, and HY: data collection. XF: writing the article. XF, ZL, ML, FF, and JW: critical revision of the article. FF and CL: obtained funding. FF: overall responsibility. All authors contributed to the article and approved the submitted version.

## Funding

This work was supported in part by the National Nature Science Foundation of China grant (82071899), the Fundamental Research Funds for the Central Universities (3332020009), and the Beijing Natural Science Foundation grant (L182067).

## Conflict of Interest

JW was employed by the company GE Healthcare. The remaining authors declare that the research was conducted in the absence of any commercial or financial relationships that could be construed as a potential conflict of interest.

## Publisher's Note

All claims expressed in this article are solely those of the authors and do not necessarily represent those of their affiliated organizations, or those of the publisher, the editors and the reviewers. Any product that may be evaluated in this article, or claim that may be made by its manufacturer, is not guaranteed or endorsed by the publisher.
