# Supplementary material for: Pre-operative Cerebral Small Vessel Disease on MR Imaging Is Associated With Cerebral Hyperperfusion After Carotid Endarterectomy
Source: Front Cardiovasc Med. 2021 Nov 18;8:734392. doi: 10.3389/fcvm.2021.734392 (PMC8636731; doi:10.3389/fcvm.2021.734392)

**Table S1.** Parameters for conventional MRI sequences.

| Sequences | T1WI | T2WI | FLAIR | DWI | T2*WI | 3D TOF MRA |
| --- | --- | --- | --- | --- | --- | --- |
| TR (ms) | 1775 | 5258 | 12000 | 3600 | 42 | 16 |
| TE (ms) | 21.6 | 84 | 122 | 64 | 23 | 2.1 |
| TI (ms) | 720 | NA | 2712 | NA | NA | NA |
| FA (°) | 111 | 142 | 160 | 90 | 20 | 20 |
| Slice thickness (mm) | 4 | 4 | 4 | 4 | 6 | 1.2 |
| Number of slices | 40 | 36 | 36 | 36 | 48 | 288 |
| Acquisition matrix | 320 × 160 | 320 × 320 | 288 × 192 | 128 × 128 | 480×288 | 256 × 256 |
| FOV (mm^2^) | 240 × 168 | 220 × 220 | 220 × 176 | 220 × 220 | 240×216 | 220 × 220 |
| b_max_ (s/mm^2^) | NA | NA | NA | 1000 | NA | NA |

T1WI, T1-weighted imaging; T2WI, T2-weighted imaging; FLAIR, fluid-attenuated inversion recovery; DWI, diffusion-weighted imaging; TOF MRA, time-of-flight MR angiography; TR, repetition time; TE, echo time; TI, inversion time; FA, flip angle; FOV, field of view.

**Table S2.** Basic data of the 16 cerebral hyperperfusion patients.

| Case | CHS | Age (years) | Gender | CAD | Symptoms | Ipsilateral stenosis | Fazekas score | Number of lacunes | Total SVD score | BP_baseline (mmHg) | BP_post_1^st^ day  (mmHg) | BP_before discharge (mmHg) |
| --- | --- | --- | --- | --- | --- | --- | --- | --- | --- | --- | --- | --- |
| 1 | Yes | 55 | Male | Yes | Stroke | Near-occlusion | 3 | 0 | 1 | 152 | **160** | 132 |
| 2 | Yes | 68 | Male | Yes | TIA | Severe | 4 | 6 | 3 | 180 | **170** | 200 |
| 3 | No | 76 | Male | Yes | None | Severe | 4 | 0 | 2 | 123 | 103 | 123 |
| 4 | No | 78 | Male | No | TIA | Severe | 6 | 4 | 4 | 137 | 145 | 131 |
| 5 | No | 57 | Male | No | TIA | Severe | 6 | 5 | 3 | 135 | 136 | 159 |
| 6 | No | 63 | Male | No | Stroke | Severe | 3 | 2 | 2 | 135 | 147 | 120 |
| 7 | No | 82 | Male | No | None | Severe | 5 | 2 | 2 | 120 | 130 | 137 |
| 8 | No | 74 | Female | Yes | Stroke | Severe | 3 | 5 | 2 | 140 | 94 | 140 |
| 9 | No | 71 | Male | No | None | Severe | 6 | 7 | 3 | 142 | 128 | 128 |
| 10 | No | 51 | Male | No | None | Near-occlusion | 1 | 0 | 0 | 165 | 130 | 159 |
| 11 | No | 68 | Male | No | None | Near-occlusion | 5 | 0 | 1 | 141 | 145 | 150 |
| 12 | No | 63 | Male | No | None | Near-occlusion | 1 | 2 | 1 | 161 | 110 | 127 |
| 13 | No | 62 | Male | Yes | None | Near-occlusion | 5 | 3 | 2 | 142 | 120 | 112 |
| 14 | No | 66 | Male | Yes | TIA | Near-occlusion | 4 | 4 | 4 | 140 | 135 | 148 |
| 15 | No | 67 | Male | No | Stroke | Near-occlusion | 2 | 1 | 2 | 132 | 130 | 125 |
| 16 | No | 62 | Male | No | Stroke | Near-occlusion | 5 | 4 | 2 | 106 | 105 | 146 |

CHS, cerebral hyperperfusion syndrome; CAD, Coronary artery disease; SVD, small vessel disease; BP_baseline, baseline systolic blood pressure on admission; BP__post_1st day_, the highest systolic blood pressure within 24h after surgery; BP_before discharge, the highest systolic blood pressure from the second day after surgery to discharge; TIA, transient ischemic attack.

**Figure S1.** Relationship between ipsilateral carotid stenosis and cerebral hyperperfusion (CH).


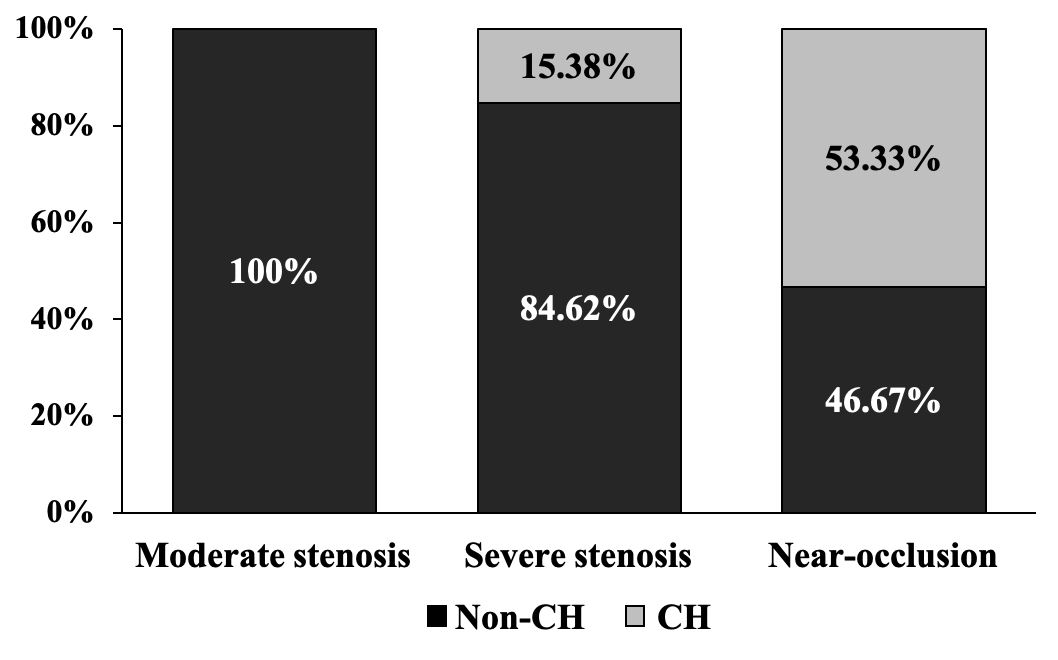

Supplement: Supplementary file 1 [file Data_Sheet_1.DOCX]
